# Understanding the perceived benefits, barriers, and cues to action for lung cancer screening among Latinos: A qualitative study

**DOI:** 10.3389/fonc.2024.1365739

**Published:** 2024-03-20

**Authors:** Edgar I. Alaniz-Cantú, Kalese Goodwin, London Smith, Eliany Acosta, Arlette Chávez-Iñiguez, Mary Jo Evans, Marcela Gaitán, Fang Lei, Reza Yousefi-Nooraie, Kevin A. Fiscella, M. Patricia Rivera, Ana Paula Cupertino, Francisco Cartujano-Barrera

**Affiliations:** ^1^ Department of Public Health Sciences, University of Rochester Medical Center, Rochester, NY, United States; ^2^ Imaging Population Health Programs, University of Rochester Medical Center, Rochester, NY, United States; ^3^ National Alliance for Hispanic Health, Washington, DC, United States; ^4^ School of Nursing, University of Minnesota, Minneapolis, MN, United States; ^5^ Department of Family Medicine, University of Rochester Medical Center, Rochester, NY, United States; ^6^ Department of Medicine, University of Rochester Medical Center, Rochester, NY, United States; ^7^ Department of Surgery, University of Rochester Medical Center, Rochester, NY, United States

**Keywords:** Latinos, Hispanics, lung cancer, lung cancer screening, lung cancer disparities

## Abstract

**Introduction:**

Rates of lung cancer screening among Latinos remain low. The purpose of the study was to understand the perceived benefits, barriers, and cues to action for lung cancer screening among Latinos.

**Methods:**

Participants (N=20) were recruited using community-based recruitment strategies. Eligibility criteria included: 1) self-identified as Hispanic/Latino, 2) spoke English and/or Spanish, and 3) met the USA Preventive Services Task Force eligibility criteria for lung cancer screening. Interviews were conducted in Spanish and English, audio recorded, and transcribed verbatim. Using the health belief model, a qualitative theoretical analysis was used to analyze the interviews.

**Results:**

Participants’ mean age was 58.3 years old (SD=5.8), half of the participants were female, 55% had completed high school or lower educational level, and 55% reported speaking more Spanish than English. All participants were currently smoking. Fourteen participants (70%) were unaware of lung cancer screening, and eighteen (90%) did not know they were eligible for lung cancer screening. Regarding lung cancer screening, participants reported multiple perceived benefits (e.g., smoking cessation, early detection of lung cancer, increased survivorship) and barriers (e.g., fear of outcomes, cost, lung cancer screening not being recommended by their clinician). Lastly, multiple cues to actions for lung cancer screening were identified (e.g., family as a cue to action for getting screened).

**Conclusions:**

Most Latinos who were eligible for lung cancer screening were unaware of it and, when informed, they reported multiple perceived benefits, barriers, and cues to action. These factors provide concrete operational strategies to address lung cancer screening among Latinos.

## 1 Introduction

Despite advances in lung cancer diagnosis and treatments, lung cancer remains the leading cause of cancer-related death worldwide, with an estimated 1.8 million deaths in 2020 (1). Among Latinos, the largest minority group in the USA (2), lung cancer accounts for a lower percentage of cases among men and women (8% and 6%, respectively) compared to men and women in the general USA population (14% and 13%, respectively) (3). This is due, in part, to the substantially younger age composition of the Latino population compared to the general USA population (4). However, the survival rates are lower among Latinos compared with non-Latino whites – making lung cancer also the leading cause of cancer-related death among Latinos (3, 5). This is partially explained by the stage at the time of diagnosis, as 83% of Latinos are diagnosed with advanced-stage disease, which carries a 5-year survival rate of only 6% (5). Increasing the complexity of the disparities faced by Latinos, lung cancer incidence and mortality are not homogeneous among Latino ethnic groups (6). For example, lung cancer incidence and mortality are nearly doubled among Cuban and Puerto Rican men compared to Mexican men (6), which reflects the disparities in tobacco use and cessation faced by some ethnic groups of Latinos (7).

Lung cancer screening with low-dose computed tomography decreases lung cancer mortality by 20% due to a stage shift, resulting in more lung cancers diagnosed at an early stage (8, 9). Despite the benefits of lung cancer screening and the strong recommendations from the USA Preventive Services Task Force and the American Cancer Society (10, 11), rates of lung cancer screening remain low (12–14). A recent study using data from 20 states that adopted comprehensive lung cancer screening programs reported an uptake rate of 20.7% (14), less than half the rate for other types of cancer screening (15). Moreover, marked disparities in the rate of lung cancer screening based on race, ethnicity, socioeconomic status, and geographic location have been reported (16–20). For example, lung cancer screening uptake among Latinos remains minimal (21). The purpose of this study was to understand the perceived benefits, barriers, and cues to action for lung cancer screening among Latinos. The information gathered from this qualitative study will inform future interventions to increase the uptake of lung cancer screening among Latinos.

## 2 Methods

### 2.1 Study design

This qualitative study consisted of semi-structured interviews in English and Spanish with Latinos to understand the perceived benefits, barriers, and cues to action for lung cancer screening. Study procedures were approved and monitored by the University of Rochester Medical Center Institutional Review Board (protocol number STUDY00007787). Participants were compensated with a $30 gift card for their time and effort.

### 2.2 Positionality

In qualitative studies, the researcher is considered a research instrument given their ability to observe details, conduct in-depth interviews, and reflect on the meaning of interview data (22). The first author, who conducted the semi-structured interviews, is a Latino medical student interested in promoting Latino health. The second and third authors, who conducted the qualitative theoretical analysis, are medical students also interested in promoting Latino health. The last author, who supervised the qualitative theoretical analysis, is a Latino researcher trained in community-based participatory research (including qualitative studies) to address disparities among Latinos (23–28).

### 2.3 Theoretical model

This study used the health belief model (HBM) as a theoretical model. The HBM posits that individuals act to change their health behaviors (e.g., getting screened for lung cancer) based on their perceived benefits, barriers, and cues to action (29). Perceived benefits refer to an individual’s opinion of the value or usefulness of a new behavior in lowering the risk of a disease and/or condition (29). Perceived barriers refer to an individual’s view of the obstacles to behavior change (29). Cues to action refer to events, people, or things that trigger people to change behavior (29).

### 2.4 Recruitment

Recruitment was conducted by a team of culturally diverse, bilingual (English and Spanish) trained recruiters between January and April 2023. Recruitment strategies included study presentations in venues with a high concentration of Latinos (e.g., community-based organizations, festivals, malls), referrals from the Wilmot Tobacco Cessation Center at the University of Rochester Medical Center, and word of mouth from community partners.

### 2.5 Eligibility

Individuals were eligible if they (1) self-identify as Hispanic/Latino; (2) speak English and/or Spanish; (3) were between 50 and 80 years old; (4) had not completed their annual screening for lung cancer with LDCT; (5) had a 20 pack-year smoking history; (6) currently smoked cigarettes or had quit within the past 15 years; (7) were interested in participating in a 90-minute interview; and (8) were comfortable with communicating over Zoom^®^ or a phone call for the interview. Eligibility criteria 3, 4, 5, and 6 were consistent with the 2021 USA Preventive Services Task Force eligibility for lung cancer screening (10). Study staff conducted an eligibility assessment over the phone in the participant’s language of preference, either English or Spanish.

### 2.6 Consent

Individuals eligible to participate in the study received an informational letter with detailed information about the study (e.g., procedures, confidentiality, contact information of the study team). The informational letter was available in English and Spanish. The first author reviewed the informative letter with participants. Verbal consent was obtained from the participants before enrollment in the study.

### 2.7 Data collection

Before the interview, the first author conducted a sociodemographic survey to collect information on age, gender, sexual orientation, education level, language of preference, and country of birth. Moreover, smoking-related variables were collected, including current smoking status, number of cigarettes smoked per day (CPD), age that the participant started smoking, time to their first cigarette, use of menthol cigarettes, quit attempt in the past year, and ever use of pharmacotherapy and electronic cigarettes for cessation. Lastly, participants were asked if they were aware of lung cancer screening (i.e., Before this study, have you ever heard about lung cancer screening with low-dose CT scan? Yes/No) and if they knew they were eligible (i.e., Before this study, did you know you were eligible to screen for lung cancer with low-dose CT scan? Yes/No). The survey was completed in the participants’ language of preference, either English or Spanish.

Immediately after the sociodemographic survey, the first author conducted a semi-structured interview. An interview guide with open-ended questions was used to facilitate the interviews. The interview was completed in the participants’ language of preference, either English or Spanish. The HBM was used to develop the interview guide (30). **Table 1** shows examples of the interview questions by the HBM factor.

**Table 1:** Examples of interview questions using the Health Belief Model.

Factor	Interview Questions
Perceived benefits	What benefits do you think an individual can get from screening for lung cancer?
Perceived barriers	What do you think may prevent an individual from screening for lung cancer?
Cues to action	What could trigger a person to decide to screen for lung cancer?

The first author conducted the interviews. All interviews were audio-taped and subsequently transcribed verbatim in the language they were conducted. Interviews in Spanish were translated to English. The interviewer verified the translations and resolved any discrepancies using a consensus approach (31).

### 2.8 Analyses

For the sociodemographic and smoking-related questions, means and frequencies were calculated. Interview data were analyzed manually after all data were collected. Qualitative theoretical analysis was used to identify, analyze, and report themes within the interview data (32). The second and third authors independently coded the first five transcripts through line-by-line coding. An iterative process was employed to achieve consensus between the two sets of codes, create a coding scheme, and develop a codebook that included details about agreed-upon code definitions. New codes were added to the coding scheme as needed until no new themes emerged with successive interviews (33). The last author supervised the qualitative theoretical analysis and provided in-depth feedback on the codebook development. All authors agreed on the final themes and sub-themes.

### 2.9 Sample size

Based on principles of data saturation, the study estimated a sample size of twenty participants to collect enough data to draw necessary conclusions (34, 35).

## 3 Results

### 3.1 Baseline characteristics

As shown in **Table 2**, the participants’ mean age was 58.3 years old (SD 5.84), 50% of the participants were female, 85% self-identified as heterosexual or straight, 55% had an education of high school or less, and 55% indicated “more Spanish than English” as their language of preference. Twelve participants (60%) were born in Puerto Rico. All participants (100%) were currently smoking, 55% smoked 1-10 cigarettes daily, 20% smoked their first cigarette within five minutes after waking up, and 70% used menthol cigarettes. Twelve participants (60%) had made a quit attempt in the past year, and 90% had used pharmacotherapy for cessation. Fourteen participants (70%) were unaware of lung cancer screening and 90% were unaware they were eligible for lung cancer screening. Half of the participants (50%) were insured by Medicaid. **Table 3** describes each participant’s age, gender, and language of preference.

**Table 2:** Characteristics of participants (n=20).

Characteristics	n = 20 (%)
Age, Mean (SD)	58.3 (5.84)
Gender	
Female	10 (50.0%)
Male	9 (45.0%)
Transgender Male	1 (5.0%)
Sexual orientation	
Heterosexual or straight	17 (85.0%)
Homosexual or gay	1 (5.0%)
Bisexual	2 (10.0%)
Education level	
High school or less	11 (55.0%)
More than high school	9 (45.0%)
Language of preference	
Only Spanish	3 (15.0%)
More Spanish than English	11 (55.0%)
Both equally	3 (15.0%)
More English than Spanish	3 (15.0%)
Country of birth	
Puerto Rico	12 (60.0%)
USA	5 (25.0%)
Cuba	2 (10.0%)
Dominican Republic	1 (5.0%)
Currently smoking	20 (100%)
Smoking pattern	
Daily, 1–10 CPD	11 (55.0%)
Daily, 11–20 CPD	9 (45.0%)
Age that participant started smoking, Mean (SD)	17.1 (3.73)
Time to first cigarette	
≤ 5 minutes after waking up	4 (20.0%)
> 5 minutes after waking up	16 (80.0%)
Use of menthol cigarettes	13 (70.0%)
Quit attempt in the past year	
Yes	12 (60.0%)
No	8 (40.0%)
Use of pharmacotherapy for cessation in the past ^1^	18 (90.0%)
Use of e-cigarettes for cessation in the past	7 (35.0%)
Unaware of lung cancer screening	14 (70.0%)
Unaware they were eligible for lung cancer screening	18 (90.0%)
Type of health insurance ^2^	
Medicaid	10 (50%)
Medicare	7 (35%)
Private	9 (45%)
Employee	2 (10%)

SD, Standard deviation; CPD, Cigarettes per day ^1^. Pharmacotherapy for cessation included the nicotine patch, nicotine gum, nicotine lozenge, nicotine nasal spray, nicotine inhaler, Chantix^®^/Varenicline, and Zyban^®^/Bupropion ^2^. Participants could have more than one insurance plan.

**Table 3:** Participants’ age, gender, and language of preference.

Participant	Age	Gender	Language of Preference
Participant 1	61	Male	More English than Spanish
Participant 2	65	Female	English and Spanish equally
Participant 3	50	Transgender male	More Spanish than English
Participant 4	61	Female	More Spanish than English
Participant 5	63	Male	More English than Spanish
Participant 6	51	Female	Only Spanish
Participant 7	74	Female	More Spanish than English
Participant 8	59	Female	Only Spanish
Participant 9	61	Male	More Spanish than English
Participant 10	62	Male	More Spanish than English
Participant 11	58	Male	More Spanish than English
Participant 12	61	Male	More Spanish than English
Participant 13	60	Female	More Spanish than English
Participant 14	52	Female	More Spanish than English
Participant 15	55	Male	More English than Spanish
Participant 16	57	Female	English and Spanish equally
Participant 17	56	Male	English and Spanish equally
Participant 18	52	Male	More Spanish than English
Participant 19	50	Female	Only Spanish
Participant 20	58	Female	More Spanish than English

### 3.2 Perceived benefits

**Figure 1:**
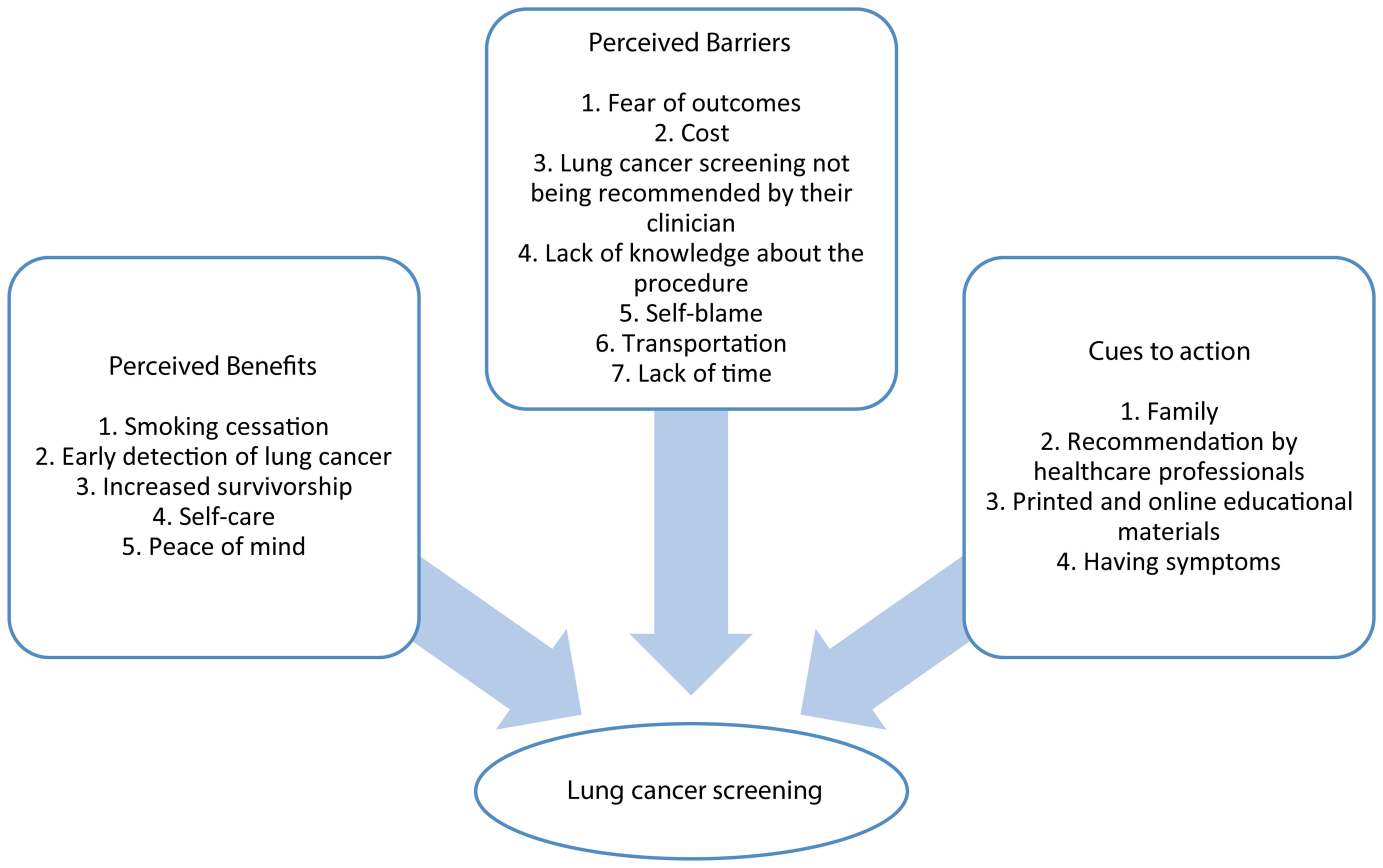
Perceived benefits, barriers, and cues to action for lung cancer screening among Latinos.

As shown in **Figure 1**, participants identified five benefits of lung cancer screening: 1) Smoking cessation, 2) early detection of lung cancer, 3) increased survivorship, 4) self-care, and 5) peace of mind. Select quotes from participants are shown below.

#### 3.2.1 Smoking cessation

Fifteen participants described that lung cancer screening would be beneficial for smoking cessation:

“It [getting screened for lung cancer] would benefit me by quitting smoking … I would stop, I would stop … It would be good because then I could try to quit smoking … The first thing that comes to mind is to completely quit smoking. And see how I would survive.” Participant 3

“Well, the benefit [of lung cancer screening] would be to know that you have a condition in your lungs and that you need to treat it … and do everything possible to turn it into a positive thing in your life, like quitting smoking”. Participant 10

“It [lung cancer screening] might also help me quit smoking … Well, maybe if I’m already sick, it would make me think and quit smoking … I’d do everything possible to quit smoking.” Participant 19

#### 3.2.2 Early detection of lung cancer

Thirteen participants described that lung cancer screening would be beneficial for early detection of lung cancer:

“I think that early detection on anything that can be fatal is great … So, it [lung cancer screening] is saving lives because of detecting it [lung cancer] early….” Participant 2

“…If it [the lung cancer] is not advanced, you have a solution … like to remove it so that it does not continue to affect the body”. Participant 5

“[A benefit would be] getting the results before it’s full-blown cancer”. Participant 13

#### 3.2.3 Increased survivorship

Nine participants described that lung cancer screening would be beneficial for increasing their survival rate:

“If I find something wrong … I would benefit because I would be in time to know … The person would be in time to make decisions or to do a treatment to save his life…” Participant 3

“If you get screened [for lung cancer] early, there’s a higher chance of living longer”. Participant 11

“There are solutions, there are things that are good, that extend life … Whether it be [lung] cancer or whatever … the [cancer] treatment lengthens your life”. Participant 18

#### 3.2.4 Self-care

Four participants described that lung cancer screening would be beneficial for self-care:

“I would take care of myself … As I told you, be more careful, prevent my lung from getting damaged completely…”. Participant 7

#### 3.2.5 Peace of mind

Three participants described that lung cancer screening would be beneficial for their peace of mind:

“[I would get screened for lung cancer] to get rid of the worry … The worry of having something…” Participant 10

### 3.3 Perceived barriers

As shown in **Figure 1**, participants identified seven barriers to lung cancer screening: 1) Fear of outcomes, 2) cost, 3) lung cancer screening not being recommended by their clinician, 4) lack of knowledge about the procedure, 5) self-blame, 6) transportation, and 7) lack of time. Select quotes from participants are shown below.

#### 3.3.1 Fear of outcomes

Seventeen participants described fear of outcomes as a barrier to lung cancer screening:

“Not having the desire to do it [getting screened for lung cancer] because of fear … Fear of being told, ‘Look, the test came back positive, and you have lung cancer. You need treatment, etc.’ Latinos are like that … We don’t want to face reality sometimes.” Participant 8

“I’m worried because I smoke, and I have a chance of getting lung cancer.” Participant 11

“Fear … That it [being diagnosed with lung cancer] could happen to me … To leave my son! I hear that word ‘cancer’ and it scares me. Fear that I have a disease…” Participant 20

#### 3.3.2 Cost

Ten participants described cost as a barrier to lung cancer screening:

“I know that financially, I don’t have the money. Because I do believe the test [lung cancer screening] is expensive … They [healthcare professionals] never told me how much it costs … I don’t know … The test is expensive, and no plan covers it”. Participant 7

“Because there are many people who their [insurance] plan does not cover them [lung cancer screening] … Or they do not have a medical plan, because then it is the lack of money.” Participant 4

“Some people don’t have the resources to get it [lung cancer screening] done because you know they charge you for everything here [at the medical center].” Participant 12

#### 3.3.3 Lung cancer screening is not being recommended by their clinician

Eight participants described lung cancer screening not recommended by their clinician as a barrier to lung cancer screening:

“Because no one, not even my doctor, ever mentioned getting a cancer screening test.” Participant 11

“To tell you the truth, they [healthcare professionals] have never recommended it [lung cancer screening] to me. I didn’t know, I found out because you called me…” Participants 18

#### 3.3.4 Lack of knowledge about the procedure

Ten participants described lack of knowledge about the procedure as a barrier to lung cancer screening:

“I’m going to be honest, I thought that the plates, X-rays, were enough. I thought that if I did the plates and no stain came out, it would be like there was nothing. I didn’t know there was another more detailed test. What is the test about? What can it prevent? Early detection? That it can save your life? Things like that … How to do it and where to?” Participant 9

“I’d like to know if it [lung cancer screening] is painful … What happens during the test and if it’s uncomfortable … I don’t know anything, I’m totally blank.” Participant 19

#### 3.3.5 Self-blame

Three participants described self-blame as a barrier to lung cancer screening:

“I mean just to know that the old bad habit [cigarette smoking], or actually my bad habit, caused me to have lung cancer … So, I did it to myself … Because nobody forced you to smoke. You do it on your own. On your own free will … Despite people telling you to quit smoking … Umm, it will have a terrible impact and I will blame myself.” Participant 13

#### 3.3.6 Transportation

Three participants described transportation as a barrier to lung cancer screening:

“Transportation is the most difficult [barrier] … Transportation … I don’t have a car” Participant 3

#### 3.3.7 Lack of time

Two participants described lack of time as a barrier to lung cancer screening:

“The lack of time … because there are many people who have two jobs, three jobs to be able to support themselves, and if they miss a day [to get screened for lung cancer] the budget is out of balance…” Participant 4

### 3.4 Cues to action

As shown in **Figure 1**, participants identified four cues to action for lung cancer screening: 1) Family, 2) recommendation by healthcare professionals, 3) printed and online educational materials, and 4) having symptoms. Select quotes from participants are shown below.

#### 3.4.1 Family

Twelve participants described family as a cue to action for lung cancer screening:

“Family is just like your own heart, man…. You can do anything as much as anybody can, you can motivate yourself [to get screened for lung cancer] just by looking at your son”. Participant 1

“My kids are always telling me to check my lungs. Because they’re always worried about how your lungs are when they see you cough or when they see you smoke. And because they care. Because they are afraid that I will leave [die] early as they say.” Participant 3

“I said to myself. Well, I want to live long enough to see my grandkids. That’s why I would do it [get screened for lung cancer] now!” Participant 13

#### 3.4.2 Recommendation by healthcare professionals

Fourteen participants described recommendation by healthcare professionals as a cue to action for lung cancer screening:

“If the doctor orders it [lung cancer screening], then I’ll get it done.” Participant 10

“Okay, if I do it, I would like my doctor to tell me ‘[Participants name] you have to stop by the [lung cancer screening] office, you have to be there…” Participant 18

#### 3.4.3 Printed and online educational materials

Fifteen participants described printed and online educational materials as a cue to action for lung cancer screening:

“Advertise the lung cancer screening program and then explain what lung cancer screening is….More information … [On a] webpage … Platforms … Social media…. Regular flyers…” Participant 1

“Have brochures in the, in the, in medical offices. In the medical buildings, in those main [medical] centers … For them to have information brochures, such as: “Get tested.” So that they explain how it is done, where it can be done, how it has to be done, that is good because people take it, and it is prevention.” Participant 4

#### 3.4.4 Symptoms

Ten participants described that having symptoms was a cue to action for lung cancer screening:

“[What could trigger a person to decide to screen for lung cancer is] the deterioration of health, the start of a period with a lot of coughing … Lack of oxygen…” Participant 9

“They [healthcare professionals] recommended that I get the lung test done because I have trouble breathing.” Participant 11

## 4 Discussion

To the best of our knowledge, this is the first qualitative study to understand the perceived benefits, barriers, and cues to action for lung cancer screening among Latinos living in the USA In this study, most Latinos were unaware of lung cancer screening (70%) and that they were eligible for screening (90%). When informed, participants identified multiple perceived benefits (e.g., smoking cessation, early detection of lung cancer), barriers (e.g., fear of outcomes, cost), and cues to action (e.g., family, recommendation by healthcare professionals) to lung cancer screening.

Percac-Lima et al. conducted a cross-sectional survey to assess barriers to and interest in lung cancer screening among 118 Latino and 342 non-Latino individuals (36). Importantly, less than half of Latinos (44.1%) were unaware of lung cancer screening (36). Moreover, findings from our study expand this work by understanding the perceived benefits and cues to action for lung cancer screening among Latinos. Nevertheless, the results of this study confirm two previously described barriers: Cost and lack of knowledge about lung cancer screening. Rodríguez-Rabassa et al. conducted focus groups with 37 individuals living in Puerto Rico to understand their perceptions of and barriers to lung cancer screening (37). Moreover, findings of this study support three previously identified barriers: 1) lack of knowledge about lung cancer screening, 2) perceived financial barriers to getting screened, and 3) fear of outcomes. In addition, our study emphasizes that if healthcare professionals recommend lung cancer screening, most Latinos would partake in the screening.

Some of the perceived barriers to lung cancer screening characterize the social determinants of health (SDOH). The SDOH are factors outside of the realm of medicine that impact health outcomes (38, 39). The SDOH include five interconnected domains: economic stability, education, neighborhood, built environment, social and community context, and access to care and health care quality (38, 39). Cost and transportation as perceived barriers to lung cancer screening are part of economic stability. Moreover, lung cancer screening not being recommended by clinicians is part of health care quality. Addressing the SDOH is imperative to make progress toward health equity, a state in which every person can attain their highest level of health (40).

Participants identified smoking cessation as a benefit of lung cancer screening. Integrating evidence-based smoking cessation treatment into lung cancer screening programs maximizes the impact of lung cancer screening (41, 42). Taylor et al. conducted a randomized controlled trial (RCT) with 818 patients receiving lung cancer screening in eight USA screening sites (43). Results of the RCT showed that delivering intensive telephone counseling and nicotine replacement therapy with lung cancer screening is an effective strategy to increase smoking cessation at month 3 (43). Moreover, in a cost-effectiveness analysis of the intervention, telephone counseling and nicotine replacement therapy were considered cost-effective in lung cancer screening settings (44). Integrating smoking cessation interventions with lung screening programs has the potential to maximize long-term health benefits at reasonable costs.

Participants identified family as a cue to action for lung cancer screening (e.g., getting screened so they can be there for their family for years to come). This result is consistent with the cultural value of *familismo* among Latinos (45, 46). *Familismo* (familism) refers to the tendency to place a high value on the central position that the family holds in the life of the individual and to view decisions by the individual in the context of the well-being of the family (45, 46). Direct encouragement among family members has been shown to influence cancer screening (e.g., breast, cervical, and colorectal cancer screening) among Latinos (47–50). Future interventions that aim to increase the uptake of lung cancer screening among Latinos should consider this identified cue to action.

### 4.1 Strengths and limitations

Our study had multiple strengths. First, this research builds upon an established history of smoking cessation research with the Latino community (23–28). Second, this work is grounded in principles of community-based participatory research (CPBR). CBPR is a partnership approach to research that involves community members, organizational representatives, and researchers across all phases of research (51). This approach ensures that the research is relevant, meaningful, and appropriate to the population for which it is planned. Third, this study benefited from an adequate sample size that gave us sufficient information to understand the perspectives of Latinos on lung cancer screening (35). Fourth, the use of the HBM framework is another strength. This model provided a conceptual framework to successfully understand identified barriers, benefits, and cues to action. Lastly, the inclusion of Spanish speaking participants is another study strength.

The study has several limitations. First, there is a possibility that participants felt compelled to offer socially desirable responses. To reduce response bias, the first author assured participants that there were no right or wrong answers before starting the interview. Second, we employed a non-probability sampling method, which limits the generalizability of the findings. Lastly, all participants were currently smoking and had medical insurance. Future studies should purposely include Latinos who have quit smoking and have no medical insurance to understand their perspectives on lung cancer screening.

## 5 Conclusion

Most Latinos who were eligible for lung cancer screening were unaware of it, and when informed, they reported multiple perceived benefits, barriers, and cues to action. These factors provide concrete operational strategies to address lung cancer screening among Latinos.

## Data availability statement

The original contributions presented in the study are included in the article/supplementary material. Further inquiries can be directed to the corresponding author.

## Ethics statement

The studies involving humans were approved by University of Rochester Medical Center. The studies were conducted in accordance with the local legislation and institutional requirements. The ethics committee/institutional review board waived the requirement of written informed consent for participation from the participants or the participants’ legal guardians/next of kin because Verbal consent was obtained from the participants before enrollment in the study.

## Author contributions

EA-C: Formal analysis, Project administration, Writing – original draft. KG: Formal analysis, Writing – review & editing. LS: Formal analysis, Writing – review & editing. EA: Data curation, Writing – review & editing. AC-I: Project administration, Writing – review & editing. ME: Writing – review & editing. MG: Writing – review & editing. FL: Writing – review & editing. RY-N: Writing – review & editing. KF: Writing – review & editing. MR: Conceptualization, Writing – review & editing. AC: Conceptualization, Writing – review & editing. FC-B: Conceptualization, Formal Analysis, Writing – original draft.
